# Supplementary material for: Current surveys may underestimate climate change skepticism evidence from list experiments in Germany and the USA
Source: PLoS One. 2021 Jul 7;16(7):e0251034. doi: 10.1371/journal.pone.0251034 (PMC8262789; doi:10.1371/journal.pone.0251034)
Supplement: S1 File — (DOCX) [file pone.0251034.s001.docx]

**S1 File for “Current Surveys May Underestimate**

**Climate Change Skepticism: Evidence from List Experiments in Germany and the USA”**

This document includes additional information regarding the research design used in the main paper. It is organized into the following sections:

1. Methods
2. Survey Procedure and Respondents
3. Experimental Design (Full Details)
4. Distribution of Item Counts
5. Balance Statistics
6. Item Wordings
7. Distribution of Respondent Characteristics

**1. Methods**

The surveys that generated the data for this study were a part of ERC Advanced Grant project no. 295456 (Sources of Legitimacy in Global Environmental Governance). Ethical approval for the project as a whole was obtained at the beginning of the project (ETH Ethics Committee approval, 28 September 2012, EK-2012-N-41, extended to March 2018). The Ethics Committee of ETH Zürich and the ERC Ethics Monitoring Unit regularly monitored the project, with the final survey items provided on a continuous basis. The surveys for this study were fielded by Ipsos, and respondents were first informed about the nature of the study before being asked for written consent. The study also followed the no-deception principle, whereby only factual information was provided to respondents.

**2. Survey procedure and respondents**

The survey was fielded with Ipsos online panels in February 2018. Fielding the survey directly with Ipsos means that they engaged in a number of quality control steps, for instance excluding speeders or preventing duplicate responders, details of which can be found at: https://ems.ipsos-mori.com/Assets/Docs/Techniques/ESOMAR-28-Questions.pdf

Ipsos used quota sampling for the survey with hard quotas based upon an individual's age, income quintile, sex, and region, and soft quotas on education and employment status. For Germany the sample size is 3620, and for the USA the sample size is 3640.

**3. Experimental Design (Full Details)**

Individuals at the beginning of the survey were asked a direct question about belief in anthropogenic climate change.

Thinking about climate change, also called global warming, which of the following statements best describes your opinion?

Randomize order of items [1] – [5]

1. Climate change is not happening.

2. Climate change is entirely caused by natural processes.

3. Climate change is partly caused by natural processes and partly caused by human activity.

4. Climate change is mainly caused by human activity.

5. Climate change is completely caused by human activity.

From which we code individuals who selected answer 1 or 2 to be disbelievers in anthropogenic climate change, i.e. that humans have no role in climate change or that climate change is not occurring.

Later on in the survey individuals took part in the list experiment. Individuals were randomly assigned to either receive the list with four “control” items, or the list with five items (four “control” items plus the sensitive item). These respective items, with the sensitive item italicized, for the USA were:

1. Raising the minimum wage to $15 would put many companies out of business
2. Repealing the Affordable Care Act (Obamacare) would harm millions of Americans
3. Banning assault weapons would reduce the murder rate in the USA
4. Trade has caused millions of Americans to lose their jobs
5. *Global warming/climate change is not caused by humans*

In Germany, these items were:

1. Wenn der Mindestlohn in Deutschland auf 12 Euro erhöht würde, würden viele Unternehmen pleite gehen.
2. Generelle Geschwindigkeitsbeschränkungen auf der Autobahn würden die Anzahl an tödlichen Unfällen verringern.
3. Freihandelsabkommen, wie das TTIP Abkommen, würden sich Produkt- und Lebensmittelstandards in Deutschland verschlechtern.
4. Durch den Atomausstieg würde der CO2 Ausstoß steigen, was der Umwelt schadet.
5. *Der Klimawandel wird nicht durch Menschen verursacht sondern ist ein natürliches Phänomen.*

translated to English these items stated:

1. Raising the minimum wage to 12 Euros would put many companies out of business
2. Adding a maximum speed limit on the Autobahn would reduce traffic fatalities
3. Free trade agreements, such as TTIP, would worsen product and food standards in Germany
4. Reducing the use of nuclear power would cause CO2 to increase and worsen the environment
5. *Global warming/climate change is not caused by humans*

With these two responses, the direct question and the item count, we estimate the proportion of individuals who do not believe in human-caused climate change using the estimator developed by Aronow et al. (2015).

**4. Distribution of Item Counts**

| **Number of Items** | **Control List (DEU)** | **Control List (DEU)** | **Control List (USA)** | **Treatment List (USA)** |
| --- | --- | --- | --- | --- |
| **0** | 0.15 | 0.15 | 0.1 | 0.08 |
| **1** | 0.28 | 0.26 | 0.25 | 0.21 |
| **2** | 0.39 | 0.33 | 0.38 | 0.35 |
| **3** | 0.14 | 0.19 | 0.18 | 0.25 |
| **4** | 0.04 | 0.05 | 0.09 | 0.07 |
| **5** |  | 0.02 |  | 0.04 |

**5. Balance Statistics**

We also check to ensure balance across our treatment conditions. Employment status is coded as a 3 point scale, with 3 equal to full time employment and 1 equal to unemployed. Education level is coded as a 3 point scale, with 3 equal to a completed college education or higher and 1 equal to no college education. As shown in Figure 1 we appear to be well balanced across treatment conditions, with no differences in means significant at the 0.05 level.

Figure S1: Balance tests. The points display values for difference in means tests of the variables mentioned, comparing the mean in the control group to those who received the sensitive item in the list experiment.

**6. Placebo Test I Estimates and P-Values**

Figure S2: Estimates and p-values for the placebo test that assesses whether any of the assumptions (Monotonicity, No Liars, No Design Effects, and Treatment Independence) are violated. Under the null hypothesis the true value of the estimate is 1, and we wish to **not** reject the null.

**Item Wordings**

*Individuals’ Income*

What is your annual household income before taxes? Please select your income category:

1. $0 - $13,234
2. $13,235 - $22,680
3. $22,681 - $32,068
4. $32,069 - $43,309
5. $43,310 - $56,025
6. $56,026 - $72,000
7. $72,001 - $90,564
8. $90,565 - $116,890
9. $116,891 - $161,915
10. $161,916 or more
11. Rather not say [SCREEN OUT AND THANK]

Wie hoch ist das ungefähre, gesamte jährliche Nettoeinkommen Ihres Haushalts? Wählen Sie bitte eine Einkommensspanne aus.

1. 11.770 € oder weniger
2. 11.771 bis 16.140 €
3. 16.141 bis 19.920 €
4. 19.921 bis 23.880 €
5. 23.881 bis 28.070 €
6. 28.071 bis 32.780 €
7. 32.781 bis 38.340 €
8. 38.341 bis 45.830 €
9. 45.831 bis 58.040 €
10. 58.041 € oder mehr
11. Möchte keine Angabe machen [SCREEN OUT AND THANK]

*Employment Status*

Which of the below best describes your employment status?

1 Full-time

2 Part-time

3 Temporarily laid off

4 Unemployed

5 Retired

6 Permanently disabled

7 Homemaker

8 Student

9 Other

Welcher Beschäftigungsstatus beschreibt Ihre aktuelle berufliche Situation am besten?

1 In Vollzeit erwerbstätig

2 In Teilzeit erwerbstätig

3 Vorübergehend freigesetzt

4 Ohne Beschäftigung

5 Im Ruhestand

6 Arbeitsunfähig

7 Hausfrau/-mann

8 Schüler(in)/Student(in)

9 Sonstiges

*Education Level*

What is the highest level of education you have completed?

1. Some education, but no high school degree
2. Graduated from high school
3. 2-year / Associate’s degree
4. Bachelor's degree
5. Master’s degree or higher

Was ist Ihr höchster Bildungsabschluss?

1. Ohne allgemeinen Schulabschluss; ohne beruflichen Abschluss
2. Hauptschul‐/Realschulabschluss/POS; ohne beruflichen Abschluss
3. Fachhochschulreife/Hochschulreife; ohne beruflichen Abschluss
4. Abschluss einer Lehrausbildung
5. Bachelorabschluss von einer Universität oder Fachhochschule
6. Diplom- bzw. Masterabschluss von einer Universität oder
7. Meister‐/Technikerausbildung oder gleichwertiger
8. Promotionsabschluss

*Sex*

What is your gender?

1. Male
2. Female

Geben Sie bitte Ihr Geschlecht an.

1. Männlich
2. Weiblich

**7. Distribution of Respondent Characteristics**

In this section we present the distribution of the respondent characteristics included in the main text: age, education, employment, income, party identification, and sex.
